# Ewing Sarcoma in the Cervical Spine Causing Left Lower Extremity Hemiparesis and Left Upper Extremity Hemiplegia: A Case Report

**DOI:** 10.5811/cpcem.47332

**Published:** 2025-12-10

**Authors:** Alexander Adler, Anne Messman

**Affiliations:** Wayne State University School of Medicine, Department of Emergency Medicine, Detroit, Michigan

**Keywords:** Ewing sarcoma, neurological examination, upper motor neuron signs, case report

## Abstract

**Introduction:**

Ewing sarcoma is a relatively common neoplasm occurring in pediatric patients 10–20 years of age, commonly presenting with bone fracture, fever, and pain and swelling at the site of the primary tumor. Here we present an unusual case of Ewing sarcoma in the cervical spine leading to neurological symptoms including left lower extremity hemiparesis and left upper extremity hemiplegia.

**Case Report:**

A 19-year-old Bengali-speaking male presented to the emergency department with a three-week history of left lower extremity hemiparesis and left upper extremity hemiplegia. Due to concern for spinal cord compression, a computed tomography of the cervical spine without contrast was obtained, which revealed a lucent lesion in the left fifth cervical (C5) vertebral body. Magnetic resonance imaging of the cervical spine revealed a left cervical extradural mass present from C3–C7. The patient subsequently underwent C3–C7 laminectomy with tumor decompression and fusion one week later. Surgical pathology revealed Ewing sarcoma. Following chemotherapy two months later the patient regained complete recovery of motor and sensory function in the left lower and left upper extremities.

**Conclusion:**

It is important for emergency physicians to broaden their differential diagnosis when the physical examination reveals neurological deficits as exhibited in this case. A broader workup must be obtained that does not solely consist of head imaging but also includes imaging of the spine to prevent missing the diagnosis.

## INTRODUCTION

Ewing sarcoma is a relatively common neoplasm occurring in pediatric patients, usually occurring between 10–20 years of age. The most common presenting symptoms include bone fracture, fever, and pain and swelling at the site of the primary tumor. The most common sites of Ewing sarcoma are the chest, legs, and pelvis, particularly around the growth plates.^1^,^2^ The cause is unknown, although accumulating evidence suggests a strong hereditary risk. The underlying mechanism in roughly 85% of cases involves a reciprocal translocation between chromosomes 11–22, which fuses the Ewing sarcoma breakpoint region 1 (EWSR1) gene of chromosome 22 to the Friend leukemia virus integration 1 (FLI1) gene of chromosome 11. The resultant chromosomal translocation leads to the translation of a new EWS-FLI1 fusion protein, which can convert usually silent chromatin regions into fully active enhancers leading to oncogenesis.^3^

The most common symptoms associated with Ewing sarcoma involve pain and swelling over the primary tumor site with intermittent fevers and other symptoms common in inflammatory systemic illness. Here we present an unusual case of Ewing sarcoma in the cervical spine leading to neurological symptoms that included left lower extremity hemiparesis and left upper extremity hemiplegia.

## CASE REPORT

A 19-year-old Bengali-speaking male presented to the emergency department (ED) with a three-week history of left-sided weakness. The history was predominantly obtained from the patient’s brother as the patient did not speak English and preferred to speak through his brother rather than through an interpreter. The patient’s brother stated that the patient had immigrated from Bangladesh several months prior to presentation and believed that he was lifting some heavy objects prior to symptom onset. Three weeks earlier the patient had developed significant difficulty moving his left arm and some difficulty walking. The symptoms had been progressing over the prior several weeks, which led him to visit his primary care doctor. The primary care doctor referred the patient to the ED out of concern for left lower extremity hemiparesis and left upper extremity hemiplegia, concerning for a cerebrovascular accident.

The patient had no known medical history. Physical examination revealed significant weakness in the left upper extremity, slight weakness in the left lower extremity, positive Babinski signs bilaterally, and a positive Romberg test. Due to the patient’s concerning neurological examination, a comprehensive metabolic panel, complete blood count, magnesium level, and computed tomography (CT) of the head and cervical spine without contrast were obtained. Neurology was consulted to evaluate the patient out of concern for potential central nervous system demyelination and spinal cord dysfunction.

**Image 1:**
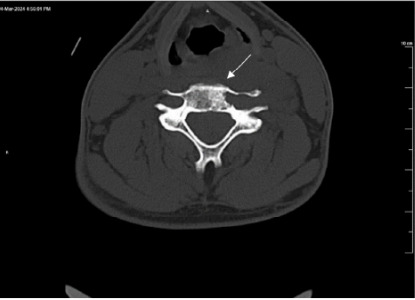
Transverse computed tomography of the cervical spine without contrast demonstrating lucent expansile lesion involving the fifth cervical vertebral body and transverse process (arrow), consistent with Ewing sarcoma.

**Image 2:**
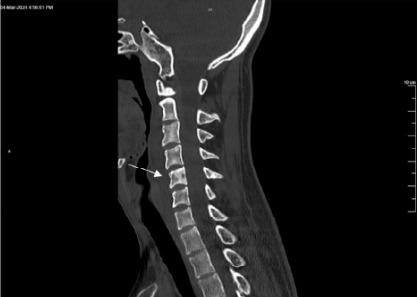
Sagittal computed tomography of the cervical spine without contrast demonstrating lucent expansile lesion involving the fifth cervical vertebral body (arrow), consistent with Ewing sarcoma.

Laboratory studies were significant for an elevated white blood cell count of 11,500 per microliter (μL) (reference range: 4,500–11,000/μL). All other lab values were within normal limits. Computed tomography of the cervical spine showed no acute findings; however, there was a lucent lesion in the left fifth cervical (C5) vertebral body extending into the transverse process (Images 1 and 2). Computed tomography of the head showed no acute intracranial hemorrhage or territorial infarct and subtle hypodensities along the bilateral precentral gyri superiorly.

**Image 3:**
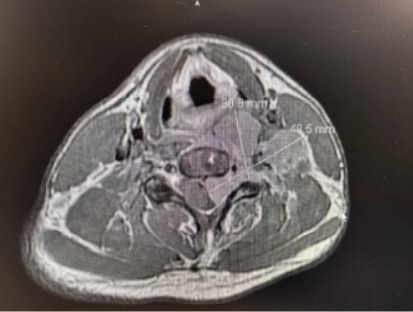
Transverse magnetic resonance imaging of the cervical spine with and without gadolinium showing dimensions of Ewing sarcoma lesion.

Neurology subsequently evaluated the patient and requested magnetic resonance imaging (MRI) of the brain and cervical spine, both with and without gadolinium, to confirm the diagnosis before admitting the patient to their service for further evaluation and treatment. The MRI of the cervical spine revealed a left cervical extradural mass present from C3–C7 with mild homogenous enhancement measuring 7.1 x 4.9 x 3.8 cm. The mass expanded into the left neural foramina and effaced the thecal sac from the level of C3–C4 to C5–C6. It caused severe spinal canal stenosis from the level of the mid-C4 vertebral body to the mid-C5 vertebral body (Image 3).

*CPC-EM Capsule*

What do we already know about this clinical entity?

*Ewing sarcoma commonly presents in patients from age 10 to 20 in the chest, legs, and pelvis.*

What makes this presentation of disease reportable?

*This case represents a unique case of Ewing sarcoma found in the cervical spine presenting with new focal neurological deficits.*

What is the major learning point?

*A complete neurological examination should be performed in patients presenting with a new onset of weakness to avoid missing the diagnosis.*

How might this improve emergency medicine practice?

*This case exemplifies the necessity of broadening the differential diagnosis to include spinal etiologies in patients presenting with focal neurological deficits.*

The patient subsequently underwent C3–C7 laminectomy with tumor decompression and fusion one week later. Surgical pathology revealed Ewing sarcoma. Following the operation the patient was admitted to the neurocritical care unit and discharged eight days later. He was instructed to return to the hematology oncology unit three days later for a multimodal chemotherapy regimen conducted as part of the Combination Chemotherapy in Treating Patients with Nonmetastatic Extracranial Ewing Sarcoma clinical trial.^4^ The patient received four cycles of chemotherapy, which he tolerated well. He was subsequently started on filgrastim for maintenance therapy. The patient was discharged approximately two months later with outpatient follow-up and a referral to radiation oncology. Physical examination conducted at the time of discharge documented complete recovery of motor and sensory function in the left lower extremity and the left upper extremity.

## DISCUSSION

This case represents an unusual presentation of Ewing sarcoma that affected the cervical spine and caused neurological symptoms consisting of hemiparesis in the left lower extremity and hemiplegia in the left upper extremity. The patient had initially complained primarily of pain and discomfort in his left shoulder; he and his brother had assumed the pain was due to overuse from increased lifting at work. Due to the persistent nature of the symptoms the patient’s primary care doctor instructed him to present to the ED for a CT head due to concern for a cerebrovascular accident.

Had the patient not received cervical spine imaging and only a CT head, as initially suggested by his primary care doctor and the neurology resident, the diagnosis would have been missed. This case highlights the importance of performing a thorough neurological examination in patients who present with weakness of any kind, and the importance of not becoming distracted by musculoskeletal complaints such as pain that often presents as the chief complaint. This patient exhibited hemiparesis in his left lower extremity and hemiplegia in his left upper extremity indicative of a focal deficit necessitating further evaluation. Additionally, he exhibited a positive Babinski sign and positive Romberg test indicative of likely spinal cord compression affecting proprioception and upper motor neuron function. This was the rationale behind adding the CT of the cervical spine, which elucidated the underlying pathology.

Although involvement of the axial skeleton by Ewing sarcoma is rare, this case illustrates the importance of including it on the differential diagnosis when evaluating patients with musculoskeletal pain and neurological deficits. Few cases have been documented in the literature. Ilaslan et al conducted a retrospective review of 1,277 cases of Ewing sarcoma, 125 of which had a primary vertebral origin. Of these, four were found in the cervical spine. It is worth noting that in all the documented cases of primary vertebral origin, localized pain was the first symptom and was seen in all the cases; neurological deficits were present in 21 cases.^5^

## CONCLUSION

Pain and swelling surrounding the primary tumor site are the cardinal symptoms of Ewing sarcoma.^6^ It is important that the emergency physician broaden the differential diagnosis in patients with these presenting complaints beyond musculoskeletal pathology and not be distracted by the patient’s chief complaint and history of present illness that may center around a potential musculoskeletal injury. This is particularly important when the physical examination reveals neurological deficits, as exhibited in this case, consistent with spinal cord compression. A broader workup must be obtained that does not solely consist of head imaging but also includes spine imaging to prevent missing the diagnosis. Additionally, it is important to keep in mind that pediatric osseous neoplasms do not always involve long bones and can involve the vertebral bodies as exhibited here.
